# Understanding the mental health of adolescents and young adults in rural South Africa through participatory research

**DOI:** 10.1371/journal.pgph.0005344

**Published:** 2025-12-12

**Authors:** Nondumiso Mthiyane, Mthobisi Zikhali, Sibongiseni Xulu, Nothando Ngwenya, Lorraine Sherr, Xanthe Hunt, Janet Seeley, Andrew Copas, Guy Harling, Maryam Shahmanesh, Thembelihle Zuma

**Affiliations:** 1 Africa Health Research Institute, KwaZulu-Natal, South Africa; 2 Institute for Global Health, University College London, London, United Kingdom; 3 University of KwaZulu-Natal, Durban, South Africa; 4 Department of Psychiatry, University of Oxford, United Kingdom; 5 South African Medical Research Council, Cape Town, South Africa; 6 Stellenbosch University, Stellenbosch, South Africa; 7 London School of Hygiene and Tropical Medicine, London, United Kingdom; 8 MRC/Wits Rural Public Health & Health Transitions Research Unit (Agincourt), University of the Witwatersrand, South Africa; University of the Witwatersrand Johannesburg Faculty of Health Sciences, SOUTH AFRICA

## Abstract

Most mental health problems in adulthood begin during adolescence. Factors such as poverty, violence, and unemployment contribute to the high burden of mental health problems. In Africa, the lack of resources for diagnosis, treatment, and management exacerbates the situation. Research on adolescent mental health has mostly focused on negative psychological effects, while positive attributes like coping, resilience, and emotional growth have been overlooked. This study explored factors affecting the mental health of adolescents and young adults and to collaboratively identify potential interventions using a participatory research approach. After reviewing the literature, we developed scenarios on issues such as unemployment, violence, poverty and HIV, which were adapted by peer supervisors into role-plays that reflect their local realities. We conducted workshops with ten peer navigators and eight young people, who listed problems affecting their mental health and identified possible interventions. Data from the workshops were synthesized to create a preliminary conceptual framework, which was then refined with input from three professional nurses who work in adolescent-friendly clinics. Young people and peer navigators identified romantic relationships and alcohol use as both contributors to mental health problems and coping strategies for stressors like poverty and violence. These behaviours were also linked to sexual and reproductive health (SRH) risks including unprotected sex. Cultural and spiritual experiences, such as the ancestral calling to become a traditional healer were also described as common in the community and were linked to emotional distress. Participants recommended family-strengthening and community-based interventions to build resilience and promote positive parenting, while respecting existing traditional practices. This study enhances our understanding of mental health challenges among young people and emphasizes the importance of strengthening protective factors such as resilience. It offers guidance for developing culturally appropriate mental health interventions. Further research is needed to examine causal pathways and explore how SRH services and traditional healing practices can be integrated into mental health strategies.

## Introduction

Mental health problems are among the leading causes of disability and mortality in adolescents and young adults [1]. In 2019, about 250 million adolescents and young adults aged 10–24 had mental disorders globally [2]. Mental disorders such as depression and anxiety may lead to severe mental health problems later in life, if not properly diagnosed, managed and treated [3]. It is well established that mental health problems in childhood and adolescence can affect adulthood experiences, and the majority of adult mental health problems first arise in childhood or adolescence [4,5]. In Africa, structural and biological factors including poverty, violence and HIV contribute to the high burden of mental health problems among adolescents and young adults [6–10]. The lack of accessible mental health care in Africa makes the problem worse, because there are not enough resources to properly identify, treat, and support people with mental health problems [11].

Adolescent mental health is a growing concern globally, with research largely focusing on negative psychological effects such as depression, post-traumatic stress disorder and anxiety [12]. However, positive mental health attributes such as coping, adjustment, resilience and emotional growth have been largely overlooked, despite being critical to overall well-being [13,14]. Emotional development, including the ability to recognize and manage emotions effectively, plays a key role in building short-term coping skills and long-term resilience [14,15]. Emotional regulation – a key aspect of emotional growth has been linked to a reduced risk of mental disorders [16–18]. As adolescents and young adults navigate changing environments and experiences including romantic relationships and experimentation and social pressures, they require resilience to adapt and thrive. In this context, resilience refers to the ability to respond positively to adversity and environmental challenges [19]. Furthermore, there is growing attention on the need to understand the social determinants of mental health [20] and the pathways through which they impact mental health.

In South Africa and across sub-Saharan Africa, the burden of mental health problems among adolescents and young adults is further complicated by the high prevalence of HIV and associated risk factors. Studies have shown a strong association between HIV exposure and poor mental health, with many young people experiencing co-occurring mental disorders and HIV-related challenges [21,22]. Mental health problems among individuals living with HIV are known to affect their quality of life, coping abilities, treatment adherence, and engagement in risk behaviours such as substance use and unsafe sexual practices [23,24]. The relationship between HIV and mental health is bidirectional and influenced by a range of intersecting social and health factors, including poverty, violence, teenage pregnancy, and other reproductive health issues [8–10,25]. These challenges heighten the vulnerability of young people to both mental disorders and HIV infection. In low- and middle-income countries (LMICs), poverty has consistently been linked to poor mental health outcomes and an increased risk of HIV. Financial insecurity, for example, can lead to chronic stress, reduced self-esteem, and greater exposure to HIV-related risk behaviours [26]. This complex interplay underscores the urgent need for integrated, context-sensitive mental health interventions that address both individual and structural risk factors affecting young people in these settings.

Emerging evidence from sub-Saharan Africa supports the value of multi-level interventions targeting individual, family, and community levels, to reduce mental health risks among vulnerable adolescents and young adults. A systematic review by Mthiyane et al. found that interventions combining economic empowerment, cognitive behavioural therapy (CBT), and peer support were effective in reducing common mental disorders in this population [27]. However, most of these interventions focused on adolescents already experiencing mental health issues, revealing a critical gap in preventive strategies. More research is needed to better understand which components make these interventions effective and how they can be tailored to specific populations and contexts. A rapid review by Coetzer et al. emphasized the importance of involving young people in the design and implementation of interventions, noting that their lived experiences and contextual knowledge significantly enhanced the relevance and success of mental health programs [28].

This study builds on existing work by using participatory methods to integrate evidence from the literature with insights from mental health service users and providers into a conceptual framework. Through a socio-ecological lens, we explore the factors contributing to poor mental health, identify potential preventive interventions, and highlight the positive changes needed to support the mental well-being of adolescents and young adults.

## Methods

### Setting

The study was conducted in the Africa Health Research Institute Demographic Surveillance System (AHRI HDSS). The AHRI HDSS is located in the north of KwaZulu-Natal, South Africa. It covers a population of approximately 140 000, including 27 000 adolescents and young adults aged 10–24 years [29]. uMkhanyakude is one of South Africa’s most underserved districts, facing high HIV prevalence and unemployment [30]. The district has five hospitals, 57 clinics (including five gateway clinics), 17 mobile clinics serving 238 stopping points and seven high-transmission-area sites. However, the implementation of National Youth-Friendly policies aimed at addressing the health needs of adolescents and young people is hindered by limited resources - both in terms of human resources for health and medical equipment, as well as structural and health system factors. Additionally, the district faces difficulties with cross-border patients due to its proximity to Mozambique and Swaziland. Many young people walk up to 45 minutes to school, as the population is spread across remote homesteads, far from essential services. Access to water, electricity, and recreational facilities is also limited. While some residents are relocating closer to towns in search of better job opportunities, this often places them farther from their designated healthcare clinics.

Between March 2018 and September 2019, AHRI developed a biosocial peer-led intervention to support youth HIV prevention - *Thetha Nami* (Talk to me) [31]. The intervention includes peer-led health promotion to improve self-efficacy and demand for HIV prevention, referrals to social and educational resources, and accessible youth-friendly clinical services to improve uptake of HIV prevention. It is from the *Thetha Nami* intervention that participants for this study were approached to participate.

### Participants and recruitment

Participants were conveniently sampled from the *Thetha Nami* project. The sample involved area-based peer navigators (n = 10), who were recruited from a pool of approximately 50 individuals actively engaged in delivering the *Thetha Nami* intervention. A team leader (responsible for line management for peer navigator supervisors) was approached to provide a list of all peer navigators and the areas in which they worked. They were recruited to provide a variation in gender, age, location and duration as a peer navigator, and were eligible if they were permanent residents in the AHRI surveillance area. Additionally, young people aged 15–24 years old (n = 8) were recruited from *Thetha Nami* intervention areas, with the help of peer navigators. These young people were randomly approached by peer navigators and given information about the study and then they were asked if they were interested in participating. Out of 10 who were approached and agreed to participate, eight were available for the workshop. Finally, professional nurses (n = 3) working in the adolescent friendly clinics were also included to provide perspectives from the clinical service delivery side. Included nurses were those in management who also had extensive prior experience in the local Department of Health.

#### Peer navigators.

Ten AHRI peer navigators reflecting a range of ages, genders, and located in different areas (rural, peri-urban and urban) were purposively invited. AHRI peer navigators are men and women aged 18–30 years, who have completed high-school and been selected by municipal and traditional authorities. They undergo a 6–8-week programme training and assessment, that includes HIV counselling and testing, HIV prevention, sexual health and youth development. Following this, they engage in supervised community-based sexual health promotion with their peers living in the intervention area [31]. All peer navigators were delivering *Thetha Nami*, and were included to share their perspectives, based on their knowledge of the study and their experiences as young people residing in the same communities where they work.

#### Young people.

After participating in the study, peer navigators were asked to invite 10 adolescents and young adults aged 15–24 years, from their communities to join the study. Each peer navigator was asked to recruit one participant from the area where they work. To assist with this, they were provided with detailed study information and instructed to share it with potential participants when extending their invitations. Young people were not recruited based on their participation in *Thetha Nami* intervention. Any young person who was approached by peer navigators was eligible to take part in the workshop.

#### Nurses.

Three highly experienced AHRI nurses who had worked in this setting with adolescents and young people for more than ten years were also invited to participate. At the time of the study, these nurses were providing clinical services in the *Thetha Nami* intervention. The nurses had experience working in both government facilities and research settings. They were chosen based on their experience of working with young people but not necessarily within the intervention area.

#### Peer navigator supervisors.

Peer navigators are young people who were first recruited as peer navigators in 2018. At the time of recruitment, they were aged 18–30-years-old and living in the communities in which they worked and had completed secondary school. They were selected through a process of referral by traditional and municipal leaderships and assessments. In 2022, eight were promoted as peer navigator supervisors to support the team leader and monitor peer support on the ground. Their role also includes day-to-day troubleshooting, and through their experience, they provide on-the-ground training of newly recruited peer navigators.

### Ethical considerations

The study was approved by the University College London (Reference number: 21831/001) and the University of KwaZulu-Natal (Reference number: BFC 515/18) Research Ethics Committees. All participants were asked to provide written consent to participate in the study. For adolescents younger than 18 years, parental consent was sought prior to participation in the study.

### Study materials and data collection

**Fig 1:**
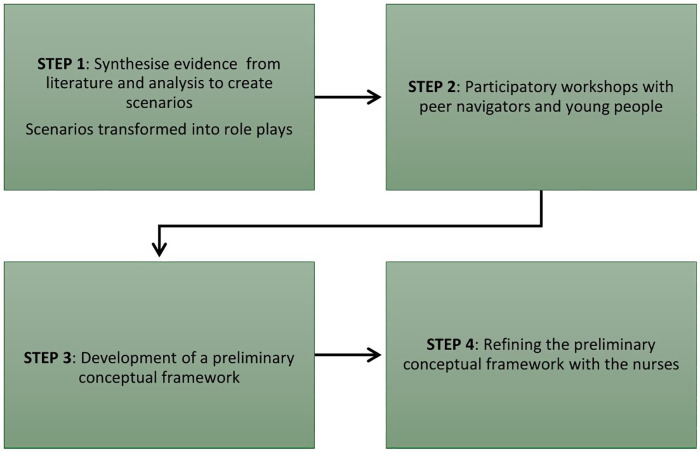
The development of a conceptual framework.

Fig 1 below outlines the five steps leading to the development and refinement of the conceptual framework.

### Literature synthesis

The literature review was conducted by the first and last authors to understand risk factors and interventions for poor mental health in adolescents. The review helped guide the development of scenarios. From the literature, social factors were identified as most common risk factors for poor mental health among adolescents and young adults in sub-Saharan Africa including our study setting (Table 1). Cognitive behavioural therapy (CBT), peer support and economic empowerment were found to be effective interventions when delivered alone or in combination with other interventions. Sustaining the long-term effects of some of these interventions was noted as a challenge that could affect their implementation. In addition, causal mechanism on how these interventions impact mental health outcomes is not well documented. [21,27,32–34].

**Table 1:** Risk factors and effective interventions for poor mental health among adolescents and young adults.

Risk factors	Interventions	Gaps/Challenges
Social factors identified in the literature and through the analysis of the study population [21,27,32–35] include: Poverty Violence Bullying HIV (stigma/affected/infected Migration Food insecurity Bereavement Pregnancy Orphanhood	Effective interventions identified in the literature review included economic empowerment, cognitive behavioural therapy and peer support [27,36,37]. Interventions were mostly implemented by non-specialists (lay community workers, teachers, etc) In this study setting in uMkhanyakude district, interventions targeting multiple vulnerabilities were delivered through DREAMS program, with limited focus on mental health [38].	*Mediating factors not described*. The studies did not report on the causal mechanism behind these effects. *Sustainability-* Some interventions failed to show effects when specific components were ceased [27].

### Scenarios describing mental health problems

Two team members (the first and last authors) developed scenarios covering a wide range of vulnerabilities (e.g., violence, poverty, unemployment, bereavement) based on literature including previous work conducted in this setting as described in Table 1. Scenarios are provided in S1 Table.

These scenarios were transformed into role-plays with the help of three AHRI trained bilingual social science research assistants (who speak isiZulu - the local language of the participants and English) and eight peer navigator supervisors (aged 18–30 years old). Peer navigator supervisors used innovative and creative methods to ensure the role-plays accurately reflected the mental health stressors faced by young people in their communities. By crafting detailed, realistic scenarios and adapting the scripts to include authentic experiences (sadness, hopelessness and lack of interest in activities), made the role-plays both engaging and reflective of real-life situations.

### Participatory workshops

Data was collected using three participatory workshops (See agenda in S1 Appendix). Data collection began on October 2, 2023, and was completed on November 7, 2023. To better understand the underlying factors contributing to mental health problems among young people and to collaboratively identify potential interventions, we conducted participatory workshops with young people, peer navigators, and nurses who work closely with this population. The participatory approach was chosen to ensure that the perspectives of those with lived experience, both young people and the professionals who support them were central to the process. This approach allowed for richer insights into the contextual and social drivers of mental health issues, while also fostering a sense of ownership and relevance in the development of potential solutions

In the beginning of each workshop, there was a general discussion with participants about mental health to gather their understanding. During these discussions, participants were asked about factors contributing to poor mental health among young people in their communities. Participatory workshops with young people and peer navigators lasted between 3–4 hours with a break between sessions, while the final workshop with nurses lasted 2 hours. Research assistants used introductory ice-breakers including team members introducing themselves, stating where they were from and mentioning one hobby. The ice breaker was done to build rapport with the research team and to help participants feel comfortable. The discussions were not audio recorded to enable participants to express themselves comfortably. Additionally, the decision not to record the workshops ensured that content not directly related to the topic of youth mental health could be summarized using a standardized template. This approach also helped create a more open environment, reducing the potential inhibiting effect of a recorder, which might have led participants to withhold important information out of fear of being recorded [39]. The use of a standardized template allowed facilitators to document key themes and insights in a focused and consistent way, while also summarizing content not directly related to youth mental health without needing to transcribe or analyse unrelated recorded material. While recording could have captured everything in full, we prioritized participant comfort and confidentiality, which could have been compromised by audio or video recording. A template with columns (problems, interventions, intermediate outcomes) was used to organize notes and simplify notetaking (see S2 Table). Research assistants were trained on capturing thorough notes.

#### Participatory workshop with peer navigators.

In the first workshop with 10 peer navigators, peer supervisors were asked to role-play how mental health affects them and other young people in the community. It was noted that scenarios played by peer supervisors could unintentionally reinforce negative stereotypes or stigmatize those with mental health conditions. Therefore, participants were given clear guidelines on how to share and discuss personal experiences while avoiding breaches of confidentiality (i.e., not mentioning names of people), and were fully informed about the purpose of the role-play as well as any potential emotional impact it might have. Role-plays were conducted in a way that promoted respect and empathy towards individuals with mental health conditions. Participating peer navigators listed common mental health problems experienced by young people in their communities and ranked each issue on a scale from 1 (least severe) to 5 (most severe), based on how significantly they believed these issues affected the young people they work with. During the discussion, peer navigators also provided possible interventions to each problem and how they would want those interventions to be delivered.

#### Participatory workshop with young people.

The second workshop with young people aged 15–24 (n = 8) was conducted a week after the first workshop. As with the first workshop, peer supervisors performed the role-plays and young people were asked to identify risk factors and solutions to poor mental health. Research assistants independently took detailed notes of what they observed and heard during the discussions. A structured template was provided to the research assistants to ensure that all relevant information was captured and that there was uniformity. Research assistants also added quotations to capture what was directly shared during discussions.

#### Developing a preliminary conceptual framework.

The two workshops were used to develop a preliminary conceptual framework to understand adolescent and young adults’ mental health problems and mediators in a South African rural setting. After each workshop, notes were collated and compared by the first author to identify differences and similarities from the documented accounts. The team then met to discuss any concerns raised by the first author. Reconciling what was heard and captured from the workshops involved a systematic approach to ensure that different perspectives and accounts were integrated effectively. The first author categorized workshop notes based on themes, topics, or issues raised. This helped in identifying common patterns or discrepancies. Areas where accounts aligned and diverged were highlighted. Mostly, noted differences were due to variations in the use of language and not on participants’ perspectives.

#### Participatory workshop with the nurses.

In the last workshop, which was held with nurses (n = 3), the researchers presented a preliminary conceptual framework and asked the nurses to identify gaps in the preliminary conceptual framework based on their experience of working with young people.

### Analysis

The analysis was based on the principles of community based participatory research (CBPR), emphasizing collaborative interpretation between participants and the study team. The first author led the analyses of data generated during discussions in the first and second workshops. The analysis was conducted using a structured approach and followed five steps: 1) In the first step, all data collected during workshop discussions was collated and compiled into one document which was organised thematically to facilitate analysis and ensure traceability of emerging themes. 2) The study team (including research assistants) had three debriefing meetings where all workshop materials were presented in order to gain a comprehensive understanding of the discussions, ideas, and themes that emerged. The team then highlighted recurring topics, issues and concepts related to mental health among young people. 3) The first author created a coding framework reflecting the identified themes and concepts and manually applied codes to segments of the workshop data compiled in step one. 4) Both first and last authors reviewed coded segments to explore relationships between themes and began to generate insights. They looked for patterns across different workshops and participant groups, and compared findings with existing literature on mental health. 5) Finally, identified themes and patterns were synthesized into a coherent framework which illustrated how different elements (e.g., risk factors, protective factors, interventions) interact within the framework. Feedback from nurses was incorporated to refine the framework, consistent with CBPR’s emphasis on community validation.

## Results

### Study participants

Of 10 peer navigators that were recruited, all took part in the participatory workshops. Of 10 young people invited to participate (five males and five females), eight attended (six males and two females) the participatory workshop. Three nurses contributed to refining the conceptual framework.

### Participatory workshops - with peer navigators and young people

In the first and second participatory workshops, participants listed and described factors that have direct and indirect impact on young people’s mental health (see S2 Appendix).

#### Risk factors.

**Romantic relationships:** Both young people and peer navigators believe that most young people get stressed by romantic relationships known locally as *umjolo,* derived from the word jolling which means having a good time. Challenges within sexual relationships, such as infidelity were highlighted as risk factors for mental health problems in young people, including those in schools. Romantic relationships were considered as the cause of mental health problems even among those without partners (i.e., those who do not have partners or get rejected by potential partners feel unworthy) as illustrated by the following quote from a young male participant:

*“If you are rejected by a girl that you want to date, it is so painful. It’s like there is something wrong with you” (Male-workshop with young people)”*

**Substance abuse:** Substance abuse was described as a mental health problem to all young people including peer navigators as demonstrated by a quote from a female peer navigator.

*“Alcohol is a problem, and it is difficult for us to intervene because we are also drinking. They will not take us seriously because we also do these things” (Female - workshop with peer navigators)*

Experimentation with sexual relationships and alcohol use among young people reflected their exploration of identity, social dynamics, and personal boundaries. Young people explained that they engaged in romantic relationships and substance use due to peer pressure and as a response to stress stemming from social problems such as poverty, lack of employment and violence in their communities. These behaviours were also characterized as risk factors for sexual reproductive health problems as they made young people to use unconventional contraceptive methods that were not clinically tested for pregnancy prevention (e.g., drinking guava leaves, swallowing marijuana seeds, burying a sanitary towel as a ritual to stop fertility, etc.). In the second workshop with young people, a male participant confirmed what was shared in the first workshop regarding mental health and substance use:

*“The reason we are dating and drinking is because we don’t have other things to do. The problems we face as young people lead to bad decisions.” (Male-workshop with young people)*

**Ancestral calling:** Peer navigators reported a rise in the number of adolescents and young adults (including those in school) who experience an ancestral calling – spiritual gift bestowed by one’s ancestors to become a traditional health practitioner [40]. They pointed out that an ancestral calling might not only affect the mental health of the individuals experiencing it, but it could also impact those around them. Those experiencing an ancestral calling were sometimes perceived as having a mental health problem and got discriminated against (in the community and schools) because of their strange behaviours or beliefs. Symptoms of an ancestral calling such as hallucinations could cause fear in other people that do not have an ancestral calling or those who do not understand how it manifests in an individual. Peer navigators also noted the challenge of engaging with young individuals who are called, as their perspectives on biomedical healthcare services may differ, potentially leading to disinterest in the services offered by peer navigators – which do not include any aspects of traditional health. This in turn meant that young people who had an ancestral calling or were perceived to have one were not benefitting from peer support.

*“Even us as peer navigators it is hard to approach someone who is a sangoma or wearing beads because their beliefs are different. They are scary sometimes..” (Female – workshop with peer navigators)*

**Violence:** Similar to what we found in the literature, young people expressed being exposed to different types of violence leading to mental problems. However, they talked more about their experiences of emotional violence as described in the quote below.

*“The teachers will shout at you in front of other learners. They assume that your marks have dropped because you are dating, while there may be other issues at home that you are dealing with” (Female - workshop with young people).*

***Violence (Bullying):*** Peer navigators also reported that bullying is common, especially among boys which sometimes escalated into serious violence such as physical altercations.

*“Boys bully each other in school, and sometimes, they end up stabbing each other. Sometimes, the bullying starts as impi yezigodi (village war) and is brought into the schools.” (Male – workshop with peer navigators)*

**Rejection from family:** Young people also mentioned that they were rejected by their families due to perceived disobedience or for their sexual identity which in turn may trigger suicidal ideation. They felt that they were misunderstood by older people as described by a male participant.

*“Some of the parents have given up on young people because of their behaviours, for some it is because of their sexuality. You always being seen as a bad child if even you have not done anything wrong,..because of your past mistakes.” (Male- workshop with young people)*

#### Suggested interventions.

Peer navigators and young people suggested mental health awareness campaigns to educate the community about different challenges (unemployment, violence, poverty etc.) and how they affect young people’s mental health.

*“There should be meetings just to educate people about the problems that we go through, even teachers should be part of these meetings.” Female – workshop with young people*

Peer navigators noted that despite the high unemployment rate, young individuals lack essential information needed to apply for jobs and pursue further education. Young people suggested career and academic initiatives aimed at aiding them with fundamental skills such as crafting a curriculum vitae or business proposal, as well as navigating the process of seeking funding to support small local businesses such as operating a car wash service. They believed that acquiring the necessary skills and receiving support from the community would aid in achieving their entrepreneurial objectives. They expressed that engaging in skills training would enhance their confidence and instil optimism about the future. A peer navigator suggested establishing support groups and a male participant recommended involving local municipal leaders:

*“We can invite young people such as those who are already in the university to be part of the support group and help those planning to attend university by sharing information.” (Female – workshop with peer navigators)*

*“If the counsellors could make sure that young people get jobs, like taking a group young people to work, even those who have given up on their lives will be motivated. They would see that they are being left behind and would want to join their peers.” (Male- workshop with young people)*

They also noted instances where fellow young people had utilized the government’s social relief grant (R350) as seed funding for their business ventures. However, they shared that some businesses failed due to a lack of business expertise, as described by a male participant:

*“I know a group of people who received the government funding to start their business, but they decided to split the money and use it for their personal things. I think the problem starts when choosing subjects in schools. People choose wrong subjects and think they can run business with those skills” (Male - workshop with young people)*

Young people pointed out recreational activities like soccer and netball tournaments as potential solutions for addressing issues related to romantic relationships and alcohol dependency. They explained that the focus on alcohol and romantic relationships comes from a lack of participating in recreational activities. They shared that engaging in sports would not only keep them occupied but also contribute to their mental well-being.

*“If there is a soccer match taking place next weekend, I’d rather go to the gym than drink or chase after girls” (Male - workshop with young people)*

Young people highlighted that witnessing their peers who have successfully overcome challenges like substance abuse and are now improving their lives through endeavours such as education and employment serves as a source of motivation for them.

*“We know a guy who was taken by an organization X to rehab, now he is back. They helped him to find a job. It is very motivating to see such things happening. If we could have a program like this, it will motivate us.” (Male - workshop with young people)*

Peer navigators also noted a challenge that might affect the delivery and impact of the suggested interventions is a lack of co-producing these interventions with the community and with young people. They emphasized the significance of accountability in interventions involving referrals and follow-ups to ensure timely access to all necessary services for young people such as victim of violence. They proposed involving traditional leaders and other esteemed members of the society in these interventions to enforce accountability measures.

*“Sometimes young people would come back to us to ask what is happening because they had not received the services that they were referred to. If izinduna (traditional leaders) are involved..because people respect them, they will make sure that they hold those people accountable.” (Male – workshop with peer navigators)*

#### Suggested mediators.

Regarding the change needed for these interventions to show impact on mental health, young people expressed the need for a supportive environment that is free of stigma and violence, one that offers economic opportunities. Such an environment would empower them to exercise their rights, including the ability to report any acts of violence perpetrated against them. Access to relevant interventions that mitigate the risk of mental health problems would build resilience among young people as described in a quote by a male participant.

*“Seeing people coming to our community to teach us about different skills motivates us. Even if we don’t get job opportunities immediately but it gives us hope” (Male – workshop with young people)*

### Development of a preliminary framework

After conducting the workshops with peer navigators and young people, we created a preliminary framework that incorporated data from the participatory workshops and the literature. The framework is divided into four sections: problems, interventions, intermediate outcomes, and impact. We categorized risk factors into behavioural and social. From the two workshops, social problems appeared to influence behavioural factors and were also a direct cause of common mental problems. The interventions were divided into two sections: prevention and treatment. Prevention interventions aimed to address behavioural and social factors while treatment such as counselling aimed to help young people who were already experiencing mental health problems. We also included intermediate outcomes which were defined as the change that is needed for these interventions to show impact on mental health. These were based on the information provided by young people and peer navigators during participatory workshops.

### Refining the conceptual framework

**Fig 2:**
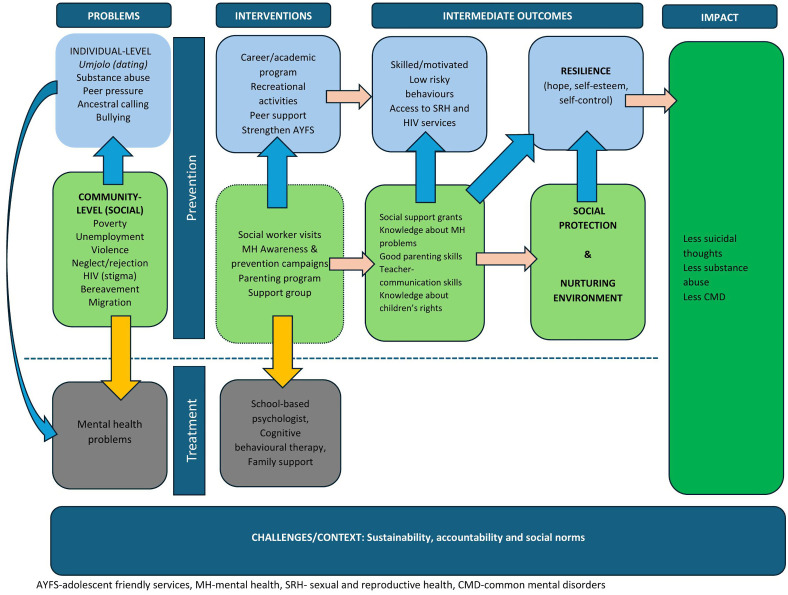
A conceptual framework.

Three professional nurses attended the third participatory workshop virtually, however, one of them could not continue with the session due to poor internet connection. The discussion with the nurses lasted for one hour. The team of researchers presented the preliminary conceptual framework based on the details provided by peer navigators and young people, which was then refined by incorporating input from the nurses, as described in Fig 2.

**Risk factors:** Regarding factors contributing to mental health problems in young people and interventions, the nurses did not suggest new information or express any disagreement with the information that was provided by peer navigators and young people.

**Suggested Interventions:** The nurses emphasized the crucial role that is played by adolescent and youth friendly clinics in the community. They shared that strengthening of adolescent and youth friendly clinics will improve access to correct sexual and reproductive health (SRH) and HIV information and services. Access to these services will lead to low unwanted pregnancies and sexually transmitted infections. They shared that youth friendly clinics are not only for providing health services, but they are like a safe space where young people are able to express their thoughts and learn.

*“What we see in our youth friendly clinics is that young people do not only come to receive health services, but they come to do their homework because of free Wi-Fi. Some accompany their friends. They end up opening up to us and asking questions, some of which are not related to health services. They get to understand that nurses are not scary people as society has painted us.” (Nurse 1)*

The nurses also agreed that creating a supportive and nurturing environment through good parenting and family support could enhance young people’s access to and utilisation of SRH/ HIV services. They highlighted issues with adherence to preventive measures such as pre-exposure prophylaxis (PrEP) and contraceptives among young people. Some youth have a fear of parental disapproval and community judgment when accessing these services. The nurses believed that if young people received support from their parents or guardians, health professionals would find it easier to deliver SRH and HIV services, potentially leading to increased adherence to SRH interventions. They proposed the idea of having a psychologist based in the school to address or manage existing mental health issues (through counselling) among young people.

*“Having a psychologist that is based in school would be very helpful, even to teachers. They (teachers) are dealing with a lot of mental health cases which they do not have the capacity to address.” (Nurse 1)*

In addition to challenges described in the preliminary conceptual framework, the nurses mentioned that some families ask for their household to be cleansed in a form of a cow to resolve rape issues within families and preserve the dignity of the families. However, this approach does not focus on the healing of the victim, as some victims do not receive counselling until they show symptoms of traumatic stress disorder later on.

## Discussion

In this study, we found that young people, and the peer navigators who support them, had a very nuanced understanding of the environment within which they live. They actively contributed to the development of a conceptual framework that integrated the existing evidence, data from the setting they live in and their own lived experience. The conceptual framework describes behavioural and social factors that contribute to mental health problems in young people and highlighted the important interaction of individual and social factors. It also recommended that tailored interventions aimed at promoting social protection and protective coping mechanism that enhance resilience to help young people adapt to and manage stressful events that lead to mental health problems.

Bullying was reported as common in schools especially among boys. These findings are consistent with previous studies conducted in South Africa and other African countries [41,42]. Bullying tends to inflict severe harm on mental health, particularly affecting vulnerable individuals including orphans who may already struggling with mental issues [22,43–47]. In contexts characterized by significant poverty, violence, and HIV prevalence, along with associated stigma, bullying among adolescents can have profound psychological effects, possibly resulting in suicide [48–50]. Bullying in schools may hinder the success of health-related initiatives addressing HIV and mental health problems, as both conditions are often stigmatised, potentially exacerbating bullying victimization [47,51]. Thus, there is an imperative for school-based health programs to integrate interventions targeting bullying in order to enhance mental health outcomes among adolescents.

Young people reported rejection by family members due to disobedience or sexuality. Rejection or neglect has serious long-term implications on young people including suicide attempts, behaviour problems and poor academic performance [52,53]. Family connections are crucial in the development of adolescents and should be a safe and non-judgemental space that allows open discussions about sexuality. In this setting, rejection by family could be influenced by cultural and societal norms as reported in the previous study by Zuma and colleagues [54], where some aspects of sexuality were considered as a taboo. Community-based programs to educate people about different aspects of sexuality and create a safe space for young people who may be experiencing rejection should be considered. Furthermore, family-strengthening interventions may improve family relationships and encourage positive parenting that facilitates the expression of one’s sexuality [55].

Peer influence among adolescents and emerging adults plays an important role in their development which involves romantic relationships. Adolescents and young adults may not possess the adaptive coping abilities that are needed to deal with stressful situations (e.g., infidelity, violence) emanating from being in a romantic relationship [56,57]. In this study, young people characterized romantic relationships as a risk factor for poor mental health not only in those engaged in romantic relationships but also among those that are not. This highlights the emotional vulnerability that is caused by peer pressure by making young people engage in romantic relationships to boosts their self-worth in the society. However, positive and supportive peer role models were identified as means to mitigate this negative effect and could be considered as part of a prevention intervention.

Similar to romantic relationships, peer pressure can cause young people especially adolescents to partake in risk-taking activities such as alcohol use. A study conducted in Uganda among adolescent girls found that peer pressure to be associated with substance abuse risky behaviour [58]. Another study conducted among South African school learners found peer pressure to be associated with drinking alcohol [59]. In settings similar to ours, marked by poverty and limited economic opportunities, alcohol and other substances may be used excessively as a way of coping with stress [60,61]. Previous studies on adults have linked heavy alcohol consumption with poverty and violence [62,63]. Therefore, family-focused and community-based interventions that encourage strong emotional and social support may mitigate the risk of unhealthy behaviours and mental health problems in young people.

Findings indicating substance abuse as a coping mechanism for stress arising from social factors align with a mixed-methods investigation conducted in Ghana by Addy and colleagues [64]. The study explored mental health difficulties and coping strategies among adolescents and revealed that while substance abuse emerged as a coping strategy, factors such as romantic relationships and spiritual influences were associated with increased mental health challenges [64]. Young people may lack strong emotional intelligence that allows them to easily adapt and deal with stressful events without relying on substance abuse, so resilience-building interventions such as peer support and CBT are needed.

Experiencing an ancestral calling was reported as a mental health problem in this study. It is not uncommon in this setting for mental health problems to be associated with ancestral calling or bewitchment [65–68]. An ethnographic study by van der Zeijst [67], conducted in rural KwaZulu-Natal found that the signs of ancestral calling are similar to mental illness symptoms and that *ukuthwasa* - the training to become a traditional healer is regarded by traditional healers as an effective treatment for mental illness. Traditional healers are regarded as an important resource in understanding and tackling mental health in rural communities. Collaboration and sharing knowledge across the different medical beliefs systems can improve mental health of young people.

This conceptual framework also highlights how mental health intersects with other health conditions such as HIV and SRH problems. As young people navigate their romantic relationships influenced by peer pressure or social factors, they face heightened risk of unwanted pregnancy and STIs transmission including HIV. Provision of HIV and SRH services alongside mental health services such as counselling may reduce the risk of HIV and mental health problems among young people. However, integrated interventions without family involvement may not be effective especially in communities lacking knowledge of mental health problems or have cultural beliefs that are against the use of SRH and HIV services by adolescence. A systematic review by Saade and colleagues reported preference for traditional treatments, stigma and lack of knowledge about mental health conditions as the most common barriers to accessing mental health interventions [69]. Therefore, there is a need to explore ways to make mental health services acceptable to the community and easily accessible to young people.

The limitation of this study is that we did not include young people with lived experience of mental health problems, whose opinions may not necessarily be the same as those of young people without such experiences. Furthermore, we did not combine participants (peer navigators, young people and nurses) in one group, although acknowledge that mixed-group discussions could have enriched the dialogue through diverse perspectives and shared learning. Nonetheless, involving young people in the discussions about their mental health and allowing them to role-play how different mental factors affect them using the language that their peers understand enhances the credibility and depth of the findings. These context-rich insights may support transferability to similar populations, particularly those with shared socio-cultural dynamics.

## Conclusion

This framework provides a better understanding of mental health issues that affect young people and underscores the importance of protective factors such as resilience to the negative influences of peer pressure and contextual factors. It can be used as guidance for developing tailored mental health interventions targeted at young people. While these interventions show promise in benefiting the mental health of youth, some may require extended periods to manifest effects. Therefore, it is crucial to explore the long-term effects of these interventions, particularly considering the heightened risks of poor mental health in this population. Further research is needed to test the causal mechanisms identified in this framework in real-life context and explore how social norms and traditional healing practices can be integrated into mental health interventions to promote positive mental health of young people.

## Supporting information

S1 Table Scenarios describing mental health problems that affect young people. (DOCX)

S2 Table Note-taking template. (DOCX)

S1 Appendix Participatory workshops Agenda. (DOCX)

S2 Appendix Participatory workshops codebook. (DOCX)

## References

[1] Global, regional, and national mortality among young people aged 10-24 years, 1950-2019: a systematic analysis for the Global Burden of Disease Study 2019. Lancet. 2021;398(10311):1593–618. 34755628 10.1016/S0140-6736(21)01546-4 PMC8576274

[2] Institute of Health Metrics and Evaluation. Global Health Data Exchange (GHDx). https://vizhub.healthdata.org/gbd-results/

[3] WHO. Adolescent mental health. Geneva: WHO. 2020. https://www.who.int/news-room/fact-sheets/detail/adolescent-mental-health

[4] Copeland WE, Wolke D, Shanahan L, Costello EJ. Adult Functional Outcomes of Common Childhood Psychiatric Problems: A Prospective, Longitudinal Study. JAMA Psychiatry. 2015;72(9):892–9. doi: 10.1001/jamapsychiatry.2015.0730 26176785 PMC4706225

[5] Schlack R, Peerenboom N, Neuperdt L, Junker S, Beyer A-K. The effects of mental health problems in childhood and adolescence in young adults: Results of the KiGGS cohort. J Health Monit. 2021;6(4):3–19. doi: 10.25646/8863 35146318 PMC8734087

[6] WHO. Adolescent and young adult health. 2021 [Available from: https://www.who.int/news-room/fact-sheets/detail/adolescents-health-risks-and-solutions

[7] Lund C, Brooke-Sumner C, Baingana F, Baron EC, Breuer E, Chandra P, et al. Social determinants of mental disorders and the Sustainable Development Goals: a systematic review of reviews. Lancet Psychiatry. 2018;5(4):357–69. doi: 10.1016/S2215-0366(18)30060-9 29580610

[8] Maharaj V, Tomita A, Thela L, Mhlongo M, Burns JK. Food Insecurity and Risk of Depression Among Refugees and Immigrants in South Africa. J Immigr Minor Health. 2017;19(3):631–7. doi: 10.1007/s10903-016-0370-x 26984226 PMC5026864

[9] Stansfeld SA, Rothon C, Das-Munshi J, Mathews C, Adams A, Clark C, et al. Exposure to violence and mental health of adolescents: South African Health and Well-being Study. BJPsych Open. 2017;3(5):257–64. doi: 10.1192/bjpo.bp.117.004861 29093828 PMC5643877

[10] Bach JM, Louw D. Depression and exposure to violence among Venda and Northern Sotho adolescents in South Africa. Afr J Psychiatry (Johannesbg). 2010;13(1):25–35. doi: 10.4314/ajpsy.v13i1.53426 20428596

[11] Docrat S, Besada D, Cleary S, Daviaud E, Lund C. Mental health system costs, resources and constraints in South Africa: a national survey. Health Policy Plan. 2019;34(9):706–19. doi: 10.1093/heapol/czz085 31544948 PMC6880339

[12] Jörns-Presentati A, Napp A-K, Dessauvagie AS, Stein DJ, Jonker D, Breet E, et al. The prevalence of mental health problems in sub-Saharan adolescents: A systematic review. PLoS One. 2021;16(5):e0251689. doi: 10.1371/journal.pone.0251689 33989357 PMC8121357

[13] Rutten BPF, Hammels C, Geschwind N, Menne-Lothmann C, Pishva E, Schruers K, et al. Resilience in mental health: linking psychological and neurobiological perspectives. Acta Psychiatr Scand. 2013;128(1):3–20. doi: 10.1111/acps.12095 23488807 PMC3746114

[14] Kim K, Lee J, Yoon J. Effects of Emotional Regulation, Resilience, and Distress Disclosure on Post-Traumatic Growth in Nursing Students. Int J Environ Res Public Health. 2023;20(4):2782. doi: 10.3390/ijerph20042782 36833480 PMC9956175

[15] Noble T, McGrath H. Emotional growth--helping children and families “bounce back”. Aust Fam Physician. 2005;34(9):749–52. 16184207

[16] Gratz KL, Roemer L. Multidimensional Assessment of Emotion Regulation and Dysregulation: Development, Factor Structure, and Initial Validation of the Difficulties in Emotion Regulation Scale. J Psychopathol Behav Assess. 2008;30(4):315–315. doi: 10.1007/s10862-008-9102-4

[17] Young KS, Sandman CF, Craske MG. Positive and Negative Emotion Regulation in Adolescence: Links to Anxiety and Depression. Brain Sci. 2019;9(4):76. doi: 10.3390/brainsci9040076 30934877 PMC6523365

[18] Schäfer JÖ, Naumann E, Holmes EA, Tuschen-Caffier B, Samson AC. Emotion Regulation Strategies in Depressive and Anxiety Symptoms in Youth: A Meta-Analytic Review. J Youth Adolesc. 2017;46(2):261–76. doi: 10.1007/s10964-016-0585-0 27734198

[19] Theron L, Ungar M. Resilience in Situational and Cultural Contexts. Handbook of Resilience in Children. Springer International Publishing. 2023:105–19. doi: 10.1007/978-3-031-14728-9_6

[20] Alegría M, NeMoyer A, Falgàs Bagué I, Wang Y, Alvarez K. Social Determinants of Mental Health: Where We Are and Where We Need to Go. Curr Psychiatry Rep. 2018;20(11):95. doi: 10.1007/s11920-018-0969-9 30221308 PMC6181118

[21] Mthiyane N, Harling G, Chimbindi N, Baisley K, Seeley J, Dreyer J, et al. Common mental disorders and HIV status in the context of DREAMS among adolescent girls and young women in rural KwaZulu-Natal, South Africa. BMC Public Health. 2021;21(1):478. doi: 10.1186/s12889-021-10527-z 33691665 PMC7945212

[22] Cluver L, Gardner F, Operario D. Poverty and psychological health among AIDS-orphaned children in Cape Town, South Africa. AIDS Care. 2009;21(6):732–41. doi: 10.1080/09540120802511885 19806489

[23] Tlhajoane M, Eaton JW, Takaruza A, Rhead R, Maswera R, Schur N, et al. Prevalence and Associations of Psychological Distress, HIV Infection and HIV Care Service Utilization in East Zimbabwe. AIDS Behav. 2018;22(5):1485–95. doi: 10.1007/s10461-017-1705-x 28194585 PMC5902521

[24] Vreeman RC, McCoy BM, Lee S. Mental health challenges among adolescents living with HIV. J Int AIDS Soc. 2017;20(Suppl 3):21497. doi: 10.7448/IAS.20.4.21497 28530045 PMC5577712

[25] Osok J, Kigamwa P, Stoep AV, Huang K-Y, Kumar M. Depression and its psychosocial risk factors in pregnant Kenyan adolescents: a cross-sectional study in a community health Centre of Nairobi. BMC Psychiatry. 2018;18(1):136. doi: 10.1186/s12888-018-1706-y 29776353 PMC5960084

[26] Frankham C, Richardson T, Maguire N. Psychological factors associated with financial hardship and mental health: A systematic review. Clin Psychol Rev. 2020;77:101832. doi: 10.1016/j.cpr.2020.101832 32088498

[27] Mthiyane N, Rapulana AM, Harling G, Copas A, Shahmanesh M. Effect of multi-level interventions on mental health outcomes among adolescents in sub-Saharan Africa: a systematic review. BMJ Open. 2023;13(10):e066586. doi: 10.1136/bmjopen-2022-066586 37788931 PMC10551963

[28] Coetzer JA, Bold A, van der Mark EJ. Exploring mental health interventions for youth in Southern Africa: A rapid review. Acta Psychol (Amst). 2022;229:103699. doi: 10.1016/j.actpsy.2022.103699 35952512

[29] Gareta D, Baisley K, Mngomezulu T, Smit T, Khoza T, Nxumalo S, et al. Cohort Profile Update: Africa Centre Demographic Information System (ACDIS) and population-based HIV survey. Int J Epidemiol. 2021;50(1):33–4. doi: 10.1093/ije/dyaa264 33437994 PMC7938501

[30] Chimbindi N, Mthiyane N, Birdthistle I, Floyd S, McGrath N, Pillay D, et al. Persistently high incidence of HIV and poor service uptake in adolescent girls and young women in rural KwaZulu-Natal, South Africa prior to DREAMS. PLoS One. 2018;13(10):e0203193. doi: 10.1371/journal.pone.0203193 30325932 PMC6191091

[31] Shahmanesh M, Okesola N, Chimbindi N, Zuma T, Mdluli S, Mthiyane N, et al. Thetha Nami: participatory development of a peer-navigator intervention to deliver biosocial HIV prevention for adolescents and youth in rural South Africa. BMC Public Health. 2021;21(1):1393. doi: 10.1186/s12889-021-11399-z 34256725 PMC8278686

[32] Dessauvagie AS, Jörns-Presentati A, Napp A-K, Stein DJ, Jonker D, Breet E, et al. The prevalence of mental health problems in sub-Saharan adolescents living with HIV: a systematic review. Glob Ment Health (Camb). 2020;7:e29. doi: 10.1017/gmh.2020.18 33489245 PMC7786273

[33] Too EK, Abubakar A, Nasambu C, Koot HM, Cuijpers P, Newton CR, et al. Prevalence and factors associated with common mental disorders in young people living with HIV in sub-Saharan Africa: a systematic review. J Int AIDS Soc. 2021;24 Suppl 2(Suppl 2):e25705. doi: 10.1002/jia2.25705 34164931 PMC8222842

[34] Mebrahtu H, Chimbindi N, Zuma T, Dreyer J, Mthiyane N, Seeley J, et al. Incident pregnancy and mental health among adolescent girls and young women in rural KwaZulu-Natal, South Africa: an observational cohort study. Int J Adolesc Youth. 2024;29(1):2371414. doi: 10.1080/02673843.2024.2371414 39035705 PMC11259029

[35] Rahman MA, Kundu S, Christopher E, Ahinkorah BO, Okyere J, Uddin R, et al. Emerging burdens of adolescent psychosocial health problems: a population-based study of 202 040 adolescents from 68 countries. BJPsych Open. 2023;9(6):e188. doi: 10.1192/bjo.2023.583 37840318 PMC10617497

[36] Coetzer JA, Bold A, van der Mark EJ. Exploring mental health interventions for youth in Southern Africa: A rapid review. Acta Psychol (Amst). 2022;229:103699. doi: 10.1016/j.actpsy.2022.103699 35952512

[37] Tareke M, Asrat Yirdaw B, Gebeyehu A, Gelaye B, Azale T. Effectiveness of school-based psychological interventions for the treatment of depression, anxiety and post-traumatic stress disorder among adolescents in sub-Saharan Africa: A systematic review of randomized controlled trials. PLoS One. 2023;18(11):e0293988. doi: 10.1371/journal.pone.0293988 37983255 PMC10659195

[38] Chimbindi N, Birdthistle I, Floyd S, Harling G, Mthiyane N, Zuma T, et al. Directed and target focused multi-sectoral adolescent HIV prevention: Insights from implementation of the “DREAMS Partnership” in rural South Africa. J Int AIDS Soc. 2020;23 Suppl 5(Suppl 5):e25575. doi: 10.1002/jia2.25575 32869481 PMC7459161

[39] Rutakumwa R, Mugisha JO, Bernays S, Kabunga E, Tumwekwase G, Mbonye M, et al. Conducting in-depth interviews with and without voice recorders: a comparative analysis. Qual Res. 2020;20(5):565–81. doi: 10.1177/1468794119884806 32903872 PMC7444018

[40] Zuma T, Wight D, Rochat T, Moshabela M. The role of traditional health practitioners in Rural KwaZulu-Natal, South Africa: generic or mode specific? BMC Complement Altern Med. 2016;16(1):304. doi: 10.1186/s12906-016-1293-8 27549895 PMC4994274

[41] Manuel D, Adams S, Mpilo M, Savahl S. Prevalence of bullying victimisation among primary school children in South Africa: a population-based study. BMC Res Notes. 2021;14(1):342. doi: 10.1186/s13104-021-05747-w 34461996 PMC8404275

[42] Aboagye RG, Seidu A-A, Hagan JE Jr, Frimpong JB, Budu E, Adu C, et al. A multi-country analysis of the prevalence and factors associated with bullying victimisation among in-school adolescents in sub-Saharan Africa: evidence from the global school-based health survey. BMC Psychiatry. 2021;21(1):325. doi: 10.1186/s12888-021-03337-5 34210264 PMC8252267

[43] Sherr L, Yakubovich AR, Skeen S, Tomlinson M, Cluver LD, Roberts KJ, et al. Depressive symptoms among children attending community based support in South Africa - pathways for disrupting risk factors. Clin Child Psychol Psychiatry. 2020;25(4):984–1001. doi: 10.1177/1359104520935502 32571077 PMC7528548

[44] Cluver L, Gardner F, Operario D. Psychological distress amongst AIDS-orphaned children in urban South Africa. J Child Psychol Psychiatry. 2007;48(8):755–63. doi: 10.1111/j.1469-7610.2007.01757.x 17683447

[45] Boyes ME, Bowes L, Cluver LD, Ward CL, Badcock NA. Bullying victimisation, internalising symptoms, and conduct problems in South African children and adolescents: a longitudinal investigation. J Abnorm Child Psychol. 2014;42(8):1313–24. doi: 10.1007/s10802-014-9888-3 24882504

[46] Owusu A, Hart P, Oliver B, Kang M. The association between bullying and psychological health among senior high school students in Ghana, West Africa. J Sch Health. 2011;81(5):231–8. doi: 10.1111/j.1746-1561.2011.00590.x 21517861

[47] Boyes ME, Cluver LD. Relationships between familial HIV/AIDS and symptoms of anxiety and depression: the mediating effect of bullying victimization in a prospective sample of South African children and adolescents. J Youth Adolesc. 2015;44(4):847–59. doi: 10.1007/s10964-014-0146-3 24996836

[48] Ashaba S, Cooper-Vince C, Maling S, Rukundo GZ, Akena D, Tsai AC. Internalized HIV stigma, bullying, major depressive disorder, and high-risk suicidality among HIV-positive adolescents in rural Uganda. Glob Ment Health (Camb). 2018;5:e22. doi: 10.1017/gmh.2018.15 29997894 PMC6036650

[49] Boyes ME, Pantelic M, Casale M, Toska E, Newnham E, Cluver LD. Prospective associations between bullying victimisation, internalised stigma, and mental health in South African adolescents living with HIV. J Affect Disord. 2020;276:418–23. doi: 10.1016/j.jad.2020.07.101 32871672

[50] Shaikh MA, Lloyd J, Acquah E, Celedonia KL, L Wilson M. Suicide attempts and behavioral correlates among a nationally representative sample of school-attending adolescents in the Republic of Malawi. BMC Public Health. 2016;16(1):843. doi: 10.1186/s12889-016-3509-8 27542733 PMC4992310

[51] Cluver L, Bowes L, Gardner F. Risk and protective factors for bullying victimization among AIDS-affected and vulnerable children in South Africa. Child Abuse Negl. 2010;34(10):793–803. doi: 10.1016/j.chiabu.2010.04.002 20880588

[52] Hovens JGFM, Wiersma JE, Giltay EJ, van Oppen P, Spinhoven P, Penninx BWJH, et al. Childhood life events and childhood trauma in adult patients with depressive, anxiety and comorbid disorders vs. controls. Acta Psychiatr Scand. 2010;122(1):66–74. doi: 10.1111/j.1600-0447.2009.01491.x 19878136

[53] Zatti C, Rosa V, Barros A, Valdivia L, Calegaro VC, Freitas LH, et al. Childhood trauma and suicide attempt: A meta-analysis of longitudinal studies from the last decade. Psychiatry Res. 2017;256:353–8. doi: 10.1016/j.psychres.2017.06.082 28683433

[54] Zuma T, Seeley J, Sibiya LO, Chimbindi N, Birdthistle I, Sherr L, et al. The Changing Landscape of Diverse HIV Treatment and Prevention Interventions: Experiences and Perceptions of Adolescents and Young Adults in Rural KwaZulu-Natal, South Africa. Front Public Health. 2019;7:336. doi: 10.3389/fpubh.2019.00336 31803703 PMC6872529

[55] Kuhn ES, Laird RD. Family support programs and adolescent mental health: review of evidence. Adolesc Health Med Ther. 2014;5:127–42. doi: 10.2147/AHMT.S48057 25177156 PMC4096456

[56] Gómez-López M, Viejo C, Ortega-Ruiz R. Well-Being and Romantic Relationships: A Systematic Review in Adolescence and Emerging Adulthood. Int J Environ Res Public Health. 2019;16(13):2415. doi: 10.3390/ijerph16132415 31284670 PMC6650954

[57] DeLong SM, Powers KA, Pence BW, Maman S, Dunkle KL, Selin A, et al. Longitudinal Trajectories of Physical Intimate Partner Violence Among Adolescent Girls in Rural South Africa: Findings From HPTN 068. The Journal of adolescent health: official publication of the Society for Adolescent Medicine. 2020;67(1):69–75. 32061464 10.1016/j.jadohealth.2019.12.016 PMC7764948

[58] Namuwonge F, Kizito S, Ssentumbwe V, Kabarambi A, Magorokosho NK, Nabunya P, et al. Peer Pressure and Risk-Taking Behaviors Among Adolescent Girls in a Region Impacted by HIV/AIDS in Southwestern Uganda. J Adolesc Health. 2024;74(1):130–9. doi: 10.1016/j.jadohealth.2023.08.006 37804302 PMC10841615

[59] Chauke TM, van der Heever H, Hoque ME. Alcohol use amongst learners in rural high school in South Africa. Afr J Prim Health Care Fam Med. 2015;7(1):e1–6. doi: 10.4102/phcfm.v7i1.755 26466397 PMC4656918

[60] Ekpenyong MS, Jagun H, Stephen HA, Bakre AT, Odejimi O, Miller E, et al. Investigation of the prevalence and factors influencing tobacco and alcohol use among adolescents in Nigeria: A systematic literature review. Drug Alcohol Depend. 2024;256:111091. doi: 10.1016/j.drugalcdep.2024.111091 38340401

[61] Machisa MT, Chirwa E, Mahlangu P, Nunze N, Sikweyiya Y, Dartnall E. Suicidal thoughts, depression, post-traumatic stress, and harmful alcohol use associated with intimate partner violence and rape exposures among female students in South Africa. Int J Environ Res Public Health. 2022;19(13). 10.3390/ijerph19137913 PMC9266174 35805572

[62] Onah MN, Field S, van Heyningen T, Honikman S. Predictors of alcohol and other drug use among pregnant women in a peri-urban South African setting. Int J Ment Health Syst. 2016;10:38. doi: 10.1186/s13033-016-0070-x 27148402 PMC4855353

[63] Andersson LMC, Twum-Antwi A, Staland-Nyman C, van Rooyen DR. Prevalence and socioeconomic characteristics of alcohol disorders among men and women in the Eastern Cape Province, South Africa. Health Soc Care Community. 2018;26(1):e143–53. doi: 10.1111/hsc.12487 28868804

[64] Addy ND, Agbozo F, Runge-Ranzinger S, Grys P. Mental health difficulties, coping mechanisms and support systems among school-going adolescents in Ghana: A mixed-methods study. PLoS One. 2021;16(4):e0250424. doi: 10.1371/journal.pone.0250424 33886671 PMC8062044

[65] Mzimkulu KG, Simbayi LC. Perspectives and Practices of Xhosa‐speaking African Traditional Healers when Managing Psychosis. International Journal of Disability, Development and Education. 2006;53(4):417–31. doi: 10.1080/10349120601008563

[66] Shange S, Ross E. “The Question Is Not How but Why Things Happen”: South African Traditional Healers’ Explanatory Model of Mental Illness, Its Diagnosis and Treatment. Journal of Cross-Cultural Psychology. 2022;53(5):503–21. doi: 10.1177/00220221221077361

[67] van der Zeijst M, Veling W, Makhathini EM, Susser E, Burns JK, Hoek HW, et al. Ancestral calling, traditional health practitioner training and mental illness: An ethnographic study from rural KwaZulu-Natal, South Africa. Transcult Psychiatry. 2021;58(4):471–85. doi: 10.1177/1363461520909615 32151201

[68] Galvin M, Chiwaye L, Moolla A. Perceptions of causes and treatment of mental illness among traditional health practitioners in Johannesburg, South Africa. S Afr J Psychol. 2023;53(3):403–15. doi: 10.1177/00812463231186264 38037643 PMC10688254

[69] Saade S, Parent-Lamarche A, Khalaf T, Makke S, Legg A. What barriers could impede access to mental health services for children and adolescents in Africa? A scoping review. BMC Health Serv Res. 2023;23(1):348. doi: 10.1186/s12913-023-09294-x 37024835 PMC10080850

