# Supplementary material for: Understanding the mental health of adolescents and young adults in rural South Africa through participatory research
Source: PLOS Glob Public Health. 2025 Dec 12;5(12):e0005344. doi: 10.1371/journal.pgph.0005344 (PMC12700379; doi:10.1371/journal.pgph.0005344)
Supplement: S1 Table — (DOCX) [file pgph.0005344.s001.docx]

S1 Table. Scenarios describing mental health problems that affect young people

| **Problem/risk factor** | **Scenario** |
| --- | --- |
| ***Poverty*** | *Lethu is 21 years old and lives with his grandmother, uncle and two sisters. The grandmother’s pension grant is their only source of income. Lethu passed his matric three years ago but has never been employed or received any training since he left high school. You notice that he seems to be drinking alcohol a little more than usual. When is not drunk, he isolates himself.* |
| ***Grief*** | *Londi is a 15 year-old girl from your village. Londi is enthusiastic and does all her school-work on time. However, as the year progresses, Londi begins to miss classes and loses focus during class activities. When you ask if everything is okay, she says she is having difficulty concentrating on her work and she is not sleeping well. Her mother passed away more than a year ago and she feels as if life is meaningless without her mother.* |
| ***Experienced violence*** | *It is a Saturday afternoon. You and your friends are planning to watch a soccer match together. However, you notice that one of your friends does not seem to be interested in anything – he just wants to be left alone. He has always been someone who loves fun and people, but recently, he has been very aggressive. When you ask him what is wrong, he tells you that he’s been physically abused by a family member, and he thinks everyone hates him.* |
| ***HIV*** | *Bongi is 17 years old. She is HIV positive and has been receiving ART from the mobile clinic. Bongi is doing matric and planning to move to a new area for her studies, but she is worried about whether she will be able to get her treatment in a new place. She thinks that she will not be accepted by the people or make new friends in a new area because of her HIV status.* |
