# Supplementary material for: Understanding the mental health of adolescents and young adults in rural South Africa through participatory research
Source: PLOS Glob Public Health. 2025 Dec 12;5(12):e0005344. doi: 10.1371/journal.pgph.0005344 (PMC12700379; doi:10.1371/journal.pgph.0005344)
Supplement: S2 Table — (DOCX) [file pgph.0005344.s002.docx]

S2 Table. Note-taking template

|  | **What is the problem/risk factors identified in the role-play?** | **What are/is the possible intervention(s) to the problem?** | **How would these interventions be delivered?** | **How will the interventions address the problem? What change is needed?** | **Other (problems, challenges, etc)** |
| --- | --- | --- | --- | --- | --- |
| **Session 1** |  |  |  |  |  |
| **Session 2** |  |  |  |  |  |
| Role-play 1 |  |  |  |  |  |
| Role-play 2 |  |  |  |  |  |
| Role-play 3 |  |  |  |  |  |
| Role-play 4 |  |  |  |  |  |
