# Supplementary material for: Understanding the mental health of adolescents and young adults in rural South Africa through participatory research
Source: PLOS Glob Public Health. 2025 Dec 12;5(12):e0005344. doi: 10.1371/journal.pgph.0005344 (PMC12700379; doi:10.1371/journal.pgph.0005344)
Supplement: S1 Appendix — (DOCX) [file pgph.0005344.s003.docx]

Lunch

Break

Break

Fig A. Agenda (Workshop 1 and 2)

Lunch

Fig B. Agenda (Workshop 3)
