# Supplementary material for: Understanding the mental health of adolescents and young adults in rural South Africa through participatory research
Source: PLOS Glob Public Health. 2025 Dec 12;5(12):e0005344. doi: 10.1371/journal.pgph.0005344 (PMC12700379; doi:10.1371/journal.pgph.0005344)
Supplement: S2 Appendix — (DOCX) [file pgph.0005344.s004.docx]

**Participatory workshops : Codebook**

| **Topics** | **Codes/labels** | **Description** | ***Example quotations*** |
| --- | --- | --- | --- |
| Risk factors | Romantic relationships | Rejection and infidelity | - *“If you are rejected by a girl that you want to date, it is so painful. It’s like there is something wrong with you” (Male-workshop with young people)* - *“If I can find out that my girlfriend has gone out with another man, ngizogowa (I’ll be stressed) straight” (Male -workshop with young people)* |
| Risk factors | Substance abuse and peer pressure | Many young people have a drinking problem – due to peer pressure  Use alcohol to fit in. | - *Alcohol is a problem, and it is difficult for us to intervene because we are also drinking. They will not take us seriously because we also do these things” (Female - workshop with peer navigators)* - *“If you don’t drink it is very hard to make friends- you become isolated” {Female – workshop with peer navigators* |
| Risk factors | Alcohol and romantic relationships | Alcohol and romantic relationships as coping mechanisms | - *“The reason we are dating and drinking is because we don’t have other things to do. The problems we face as young people lead to bad decisions.” (Male-workshop with young people)* |
| Risk factors | Ancestral calling | Influence of social norms/beliefs on mental health | - *“There is an increasing number of young people especially girls who are undergoing training to become traditional healers– for some this manifest as ukuhayiza (screaming) during school assemblies.” Male- workshop with peer navigators* - *“Even us as peer navigators it is hard to approach someone who is a sangoma or wearing beads because their beliefs are different. They are scary sometimes..” (Female – workshop with peer navigators)* |
| Risk factors | Violence | Experiencing violence within families and in schools | - *“The teachers will shout at you in front of other learners. They assume that your marks have dropped because you are dating, while there may be other issues at home that you are dealing with” (Female - workshop with young people).* - *“Some of the parents have given up on young people because of their behaviours, for some it is because of their sexuality. You always being seen as a bad child if even you have not done anything wrong, ..because of your past mistakes.” (Male- workshop with young people)* |
| Risk factors | Violence (Bullying) | Experiencing violence by peers including emotional violence | - *“Boys bully each other in school, and sometimes, they end up stabbing each other. Sometimes, the bullying starts as impi yezigodi (village war) and is brought into the schools.” (Male – workshop with peer navigators)* - *“If you are doing well in school, and your friends want you to do other things with them, they would call you names and say you are arrogant if you tell them that you are busy studying,.” (Male, workshop with young people)* |
| Risk factors | Poverty | Low self-esteem as a result of poverty | - *“Other young people have a problem of not accepting their living conditions. You find that they even lie and don’t want other people to know where they live.“ (Male, workshop with young people)* |
| Risk factors | Lack of economic opportunities | Completing school and not finding employment/getting trained | - *“We end up having suicidal thoughts because we are unable to achieve our goals.” Male- workshop with young people* |
| Risk factors | Emotional abuse | Teacher-child communication needs to improve | - *“It is not easy for school children to talk about their problems- someone has to reach out to them” Female – workshop with peer navigators* |
| Risk factors | Emotional abuse | Parenting problems | - *“The uncle is complaining about him not being employed and useless, yet himself is also unemployed. It may happen that he is also stressed” Male - Workshop with young people* |
| Risk factors | Romantic relationships | Being in a romantic relationship (e.g., dealing with partner infidelity) can cause stress in young people. | - *“Most young people get stressed by umjolo. The information that they share with us is different from the data that you collect from them. We know that they are dating” (Female- workshop with peer navigators)* |
| Intervention | Social worker | Social worker from a private organisation to identify young people with social needs | - *“Government social workers are already overloaded. It is better if the service provider is not from the same community- especially when dealing with sensitive matters.” Female – workshop with peer navigators* |
| Intervention | Community mobilisation/ Mental health awareness | Involve religious and community leaders to address social issues | - *“I think most of these problems can only be addressed at a community level. Religious leaders especially from the church X where most people (especially men) in this community attend, should be involved.” Nurse 1* |
| Intervention | Parent/caregiver involvement | Young people do not use SRH services because they are scared of their family members/caregivers which put them at risk of HIV and other health conditions that affect their mental health | - *“It would be very helpful to have parents involved when it comes to the health of their children. It will make things easy even for us as health care providers, because you find that they(young people) stop using the services such as PrEP or contraceptives because they are scared of their parents.” Nurse 1* |
| Intervention | School-based psychologist | Teacher can’t handle mental health issues – they are already dealing with a lot of problems in schools | - *“Having a psychologist that is based in school would be very helpful, even to teachers. They (teachers) are dealing with a lot of mental health cases which they do not have the capacity to address.” Nurse 1* |
| Intervention | Education/training | Peer support | - *“We can invite young people such as those who are already in the university to be part of the support group and help those planning to attend university by sharing information.” Female – workshop with peer navigators* |
| Mediators | Hope and resilience | Participating in training give them hope | - *“We know a guy who was taken by an organization X to rehab, now he is back. They helped him to find a job. It is very motivating to see such things happening. If we could have a program like this, it will motivate us.” (Male - workshop with young people)* - *“Seeing people coming to our community to teach us about different skills motivates us. Even if we don’t get job opportunities immediately but it gives us hope” (Male – workshop with young people)* |
| Intervention/  challenges | Parenting program | Existing parenting programs are not effective | - *“They would put them (parents and a child) in one room and it is difficult for a child to report their parents in their presence, and the child would end up not reporting them” Female – workshop with peer navigators* |
| Intervention | Recreational activities | Young people do not participate in any sport and end up engaging in risk behaviours | - *“If there is a soccer match taking place next weekend, I’d rather go to the gym than drink or chase after girls” (Male - workshop with young people)*   *“I have been working and living in this community for many years and agree that there is no sport ground where young people can play to release stress” Nurse 2* |
| Intervention | Economic empowerment | Economic subsidies and training support | - *“I know a group of people who received the government funding to start their business, but they decided to split the money and use it for their personal things. I think the problem starts when choosing subjects in schools. People choose wrong subjects and think they can run business with those skills” (Male - workshop with young people)* |
| Intervention | Strengthening adolescent young friendly services | Facilitate easy access to SRH services and information | - *“What we see in our youth friendly clinics is that young people do not only come to receive health services, but they come to do their homework because of free Wi-Fi. Some accompany their friends. They end up opening up to us and asking questions, some of which are not related to health services. They get to understand that nurses are not scary people as society has painted us.” (Nurse 1)* |
| Intervention | Economic empowerment/job opportunities | Involving local municipal leaders – to create job opportunities for young people. | - *“If the counsellors could make sure that young people get jobs, like taking a group young people to work, even those who have given up on their lives will be motivated. They would see that they are being left behind and would want to join their peers.” Male- Workshop with young people* |
| Intervention | Mental health awareness campaigns to educate people about mental health | Older people do not understand how youth is affected by poverty, unemployment and violence (including emotional abuse by parents, teachers and other family members). | - *“There should be meetings just to educate people about the problems that we go through, even teachers should be part of these meetings. Female – workshop with young people”* |
| Challenges | Accountability | Involvement of community leaders and a role of men in parenting. Men are not actively involved in parenting | - *“If traditional leaders are part of these meetings, even men will attend because they respect them.” Male – workshop with peer navigators* |
| Challenges | Intervention delivery and accountability | Lack of accountability affect the effectiveness of the intervention | - *“Sometimes young people would come back to us to ask what is happening, because they had not received the services that they were referred to. If the izinduna (traditional leaders) are involved ..because people respect them, they will make sure that they hold those people accountable” (Male – workshop with peer navigators)* |
| Context | Social norms/culture | Some of the culture/ beliefs may influence how healthcare interventions are received and may pose challenges to the implementation of interventions. | - *“We know cases where young people’s mental health is not considered. For example, there is a case where a girl was raped but the issue was resolved by both families. Some families would just pay a cow to the family of a victim. The problem with this is that they do not consider the trauma that the child has experienced and its impact. The child has moved to another town but is not well due to mental health problems.” Nurse 1* |
